# Medical knowledge decline: the role of active usage

**DOI:** 10.1007/s10459-025-10461-4

**Published:** 2025-07-30

**Authors:** Yunting Liu, Yanlin Jiang, Andrew D. Dallas, Mirela Bruza-Augatis

**Affiliations:** 1 Berkeley School of Education, University of California, Berkeley, CA USA; 2 School of Public Health, University of California, Berkeley, CA USA; 3 The National Commission on Certification of Physician Assistants, Atlanta, GA USA

**Keywords:** Clinical competence, Maintenance of Certification (MOC), Knowledge retention, Latent transition analysis, Longitudinal Assessment, Continuing medical education (CME)

## Abstract

This paper details the reason for the decline in medical knowledge after initial certification of physician assistants/associates (PAs) and suggests improvement in competency assessment after initial certification. We hypothesized that the decline was caused by less frequency of use; in other words, knowledge retention was impacted by the active use of knowledge. If so, the likelihood of a decline in knowledge is mediated by the closeness of the test content to the practitioners’ daily practice. Data from Physician Assistants (PA) initial certification (PANCE) and re-certification (PANRE-LA, after 6 years) were used for the current study. To quantify the level of active usage, knowledge subdomains were classified into three categories for each medical specialty: dominant, relevant and distant, ranging from the most frequently used to the least used knowledge, which was verified by four independent board-certified PAs with clinical and educational experience. To test the hypothesis, Latent transition analysis (LTA) is used to measure the probability of transitions among behavioral patterns over time, in particular how various levels of transition probabilities (e.g., probability from proficient switching to non-proficient) are related to the frequency of use. We found that the trends of knowledge decline are influenced by practice profile (medical specialty), mainly, knowledge active in daily use (i.e., dominant knowledge) over time— the less frequent the knowledge is used, the more likely the knowledge decline will take place. In particular, compared to dominant knowledge (i.e., most frequently used knowledge), relevant knowledge (i.e., mediumly frequent used knowledge) and distant knowledge (i.e., rarely used knowledge) are more likely to decline (OR = 2.31, CI = [1.82, 2.94], *p* < 0.001; OR = 2.26, CI = [1.84, 2.78], *p* < 0.001). Moreover, dominant system knowledge has a better chance to improve over the years as compared to relevant and distant system knowledge (OR = 2.19, CI = [1.71, 2.81], *p* < 0.001; OR = 2.12, CI = [1.72, 2.65], *p* < 0.001). Instead of a uniform knowledge decay, medical practitioners suffer from a differential likelihood of knowledge decay over different systems knowledge. Implications for re-certification exams are discussed.

## Introduction

Prior research shows that there is a performance decline in certifying exams after initial certification, or as physicians move further away from formal training. For example, a longitudinal study demonstrated that family medicine physician performance on recertification exams declines with time in practice (Leigh et al., 1993). A cross-sectional study also showed that time since initial certification was negatively related to performance on the certification exam. Ramsey et al. (1991) caution that some physicians might not be keeping up with the current knowledge, further strengthening the necessity to carry out recertification periodically after initial certification. The reason behind the decline might be multifold. First, after initial certification, physicians start to settle into specific practice patterns, their scope of practice often narrows, leading to reduced engagement with the full breadth of medical knowledge assessed on certification exams. This narrowing may limit opportunities to reinforce and update knowledge in less frequently encountered domains. Zhang (2016) used a hierarchical linear model for the proficiency change on the physician assistant/associate (PA) recertification exam over time and found that the decline was associated with the specialty of the PA and PAs who practice in general medicine (such as family medicine, internal medicine, etc.) demonstrated less deterioration compared to their peers. Peterson et al. (2015) also argued that the broader the scope of practice (i.e., how commonly physicians use this knowledge) would be the major impact factor of the knowledge decline. The authors found that the variability of clinical activities a physician can successfully account for the difference in passing rate for different practice profiles (medical specialties). Secondly, physicians in clinical practice operate under a significant cognitive load, balancing patient care, administrative tasks, and time pressures. According to Cognitive Load Theory (CLT), when load exceeds capacity, physicians may struggle to integrate new knowledge or reflect on practice (Szulewski et al., 2021), partially explaining for the knowledge decline.

Meanwhile, another line of research suggests that the decline in knowledge with time is not universal. This is supported by evidence that the recertification group has a higher mean proficiency score than the initial certification group when both are assessed on the same scale. Yet, despite this higher average performance, the recertification group has a lower pass rate, even though the same passing score is applied to both groups (Peabody et al., 2015). The authors argued that their holistic knowledge does improve over time, but as physicians become more seasoned, their scope of practice is more limited, and thus they lose familiarity with other domains. They tend to only update knowledge in areas of greater interest to them or relevant to clinical practice. A potential reason is that, according to dual processing theory, physicians often rely on fast, intuitive (System 1) reasoning built from experience in their routine practice, and only when deliberate reflection or reasoning takes place, the analytical (System 2) will be activated (Norman et al., 2017). While system 1 is useful, it does not support learning and might even reinforce bias (Croskerry, 2009). On the contrary, system 2 might bring room for improvement and learning (Mamede et al., 2007), which often happens in cases related to their specific medical practice (specialty). As a result, working knowledge may deteriorate in areas outside their practice (specialty) and improve in their own practice (specialty). O’Neil and Puffer (2013) showed that knowledge can be maintained or even become more advanced than that of recent graduates as long as necessary practice and mandatory assessment are implemented. In fact, when reexamination is required for diplomates, they actually perform better than recent graduates, as well as those diplomates who have gaps in their recertification.

Therefore, given the commonly perceived decline in knowledge and contradictory findings on some certification maintenance scores, a natural question would be whether the decline is limited to a certain range and thus varies in detectability in tests designed with different blueprints. As cognitive psychology suggests, active use of knowledge is beneficial for knowledge encapsulation and retention (Schmidt & Rikers, 2007). As different medical specialties involve a different range of challenges, various practice profiles might suffer from different rates of decline according to how “active” the knowledge is activated in practice (CITE). To quantify the level of active usage, knowledge subdomains can be classified according to their daily practice (e.g., different levels of active usage).

In this work, we aim to answer the following research questions: Acknowledging the decline between initial certification and recertification, what impact do medical specialties bring in the decline? More importantly, if medical practitioners practicing in different specialties show different patterns of change, can we explain that decline by their frequency of using knowledge in certain domains?

## Methods

### Instruments and data

To become a board-certified physician assistant/associate (PA-C) in the United States (US), individuals must complete a multi-step process governed by the National Commission on Certification of Physician Assistants (NCCPA). After graduating from an accredited PA program, they must pass the Physician Assistant National Certifying Examination (PANCE), which serves as the initial certification exam. To maintain certification, PAs must recertify every 10 years (or 6 years according to the older rules before 2014 PANCE cohort) by passing either the Physician Assistant National Recertifying Examination (PANRE) or the PANRE Longitudinal Assessment (PANRE-LA); otherwise, they will lose their certification status. All exams assess clinical knowledge, reasoning, and medical professional practice.

PANCE is a 300-question multiple-choice exam consisting of five blocks of 60 questions, with 60 min per block. All questions are from a “common core” defined by the examination test plan^1^. A description of the content categories, the percentage of items assigned to each category, and the rationale for using these categories and percentages are well documented^2^. PANRE is administered at a secured testing center at one point in time and includes a 240-question multiple-choice exam, whereas PANRE-LA is administered in 12 quarters over 3 years, with 25 questions quarterly. PANRE-LA promotes self-directed learning by identifying and addressing knowledge gaps in core medical content.

The 2013 PANCE cohort was used to answer the current study questions. These PANCE takers took the PANRE-LA pilot test in 2019 and 2020 (*n* = 3,139). Demographic information was collected when they took the recertification exam, including self-reported medical specialties, practice US state, age, gender, and race. Table 1 presents demographic characteristics of the study sample. Since there are, in total, 64 possible practice profile (medical specialties), we did not detail how many PAs practice under each category. Instead, we show how many are in multi-specialization (can be interpreted as general specialization, e.g., family medicine, emergency medicine, internal medicine, etc.) and single specialization.

**Table 1:** Descriptive statistics for 2013 PANCE cohort entering PANRE-LA exam (*N* = 3,139)

Practice Profile	
Multi-specialization	1,514(48%)
Single specialization	1,625(52%)
Gender	
Male	796(25%)
Female	2,343(75%)
Race (Top 3 listed)	
White	2,578(82%)
Asian	194(6%)
Black/African American	57(2%)
Age Group at recertification (Top 5 listed)	AgeGroup2018
25–29	29(1%)
30–34	1,744(56%)
35–39	889(28%)
40–44	270(9%)
45–49	105(3%)

### Characterizing frequency of use

In order to study how PAs’ medical specialty impacts their knowledge retention rate, especially to test the hypothesis of whether active usage strengthens knowledge retention, we label knowledge according to their frequency of use for PAs in different medical specialty, respectively.

The medical specialty is reported by the PAs at the beginning of the exam (e.g., PANRE). In total, 64 practice profiles (medical specialties) can be chosen, such as addiction medicine, anesthesiology, critical care medicine, dermatology, etc. One person can only select one medical specialty. The grouping of knowledge follows the exam blueprint listed on the NCCPA website, which includes 14 system knowledge – cardiovascular, dermatologic, endocrine, eyes/ears/nose/throat, gastrointestinal/nutrition, genitourinary, hematologic, infectious diseases, musculoskeletal, neurologic, psychiatry/behavioral science, pulmonary, renal, and reproductive systems. Each of system knowledge typically refers to knowledge about one or several organ systems. For this study, we used four types of labels to classify how frequently PAs in certain medical specialty use the system knowledge:

- Reference: Many PAs do not develop a medical specialization since their practice belongs to general practice (or Multi-specialization in Table 1, e.g., family medicine, emergency medicine, internal medicine). Therefore, all system knowledge are considered equally important and labeled “reference” when the practice profile (medical specialty) is general practice.
- Dominant: PAs use a specific system knowledge most commonly. For example, neurologic system knowledge is a dominant system knowledge for PAs who practice in neurological surgery.
- Relevant: PAs may often use this system knowledge in their practice, yet it is not the core knowledge (dominant) for their specialty. In this case each practice (specialty) may have up to 2 relevant knowledge domains. For example, PAs who practice in neurological surgery, as a medical specialty, may have two relevant system knowledge – cardiovascular and endocrine system.
- Distant: PAs will still use this system knowledge but might be a bit unfamiliar with it (e.g., need consultation), as it is knowledge they may not use in daily practice as part of their primary specialty. All other systems not labeled as dominant or relevant are defined as distant, so we do not need to specify this cluster. For example, for PAs who practice in neurological surgery, system knowledge that are not the neurologic system (dominant), cardiovascular and endocrine systems (relevant) are classified as distant system knowledge (e.g., psychiatry/behavior science).

The above labeling was first created by two of the paper’s authors (one of whom has PA experience and has served as a PA educator), and then validated by three external, experienced PAs (also PA educators) who were blinded to the purpose of the study. Then, to answer the research question, for each label (dominant, relevant, distant, reference), the behavior is modeled together. In the next section, we will elaborate on how we use a transition matrix inspired by a Markov chain to model the change.

### Latent transition analysis

Markov chain models have been used to analyze individual growth over time and describe different kinds of stagewise development (Kaplan, 2008; Hickendorff et al., 2018). Latent transition analysis (LTA) is intended to measure dynamic latent variables, which are assumed to transition through a series of latent stages over time (Graham et al., 1991). The patterns of movement between latent classes are modeled by a stochastic process characterized by a Markov chain (Cho et al., 2010). Since we only have two time points – the initial certification and the first recertification, we will only rely on this information to calculate the state transition probabilities. Thus, there are four possible change patterns for two time points, namely:

- From Proficient to Proficient (Knowledge maintenance, denote as (1,1)). This is the level that an ideal PA belongs to, during years of practice, his/her knowledge did not decline significantly in this area. What kind of knowledge gets best preserved and updated is of our primary interest.
- From Proficient to Non-proficient (Knowledge decline, denote as (1,0)). This type of change is of particular interest, as we wonder what kind of subdomain will suffer this significant decline in knowledge, and what the subdomains’ relationship with the PA’s practice over years.
- From Non-proficient to Proficient (Knowledge improvement, denote as (0,1)). During years of practice, the PA’s knowledge significantly improved in this subdomain.
- From Non-proficient to non-proficient (denote as (0,0)). The knowledge in this domain was consistently below average over time.

**Fig. 1:**
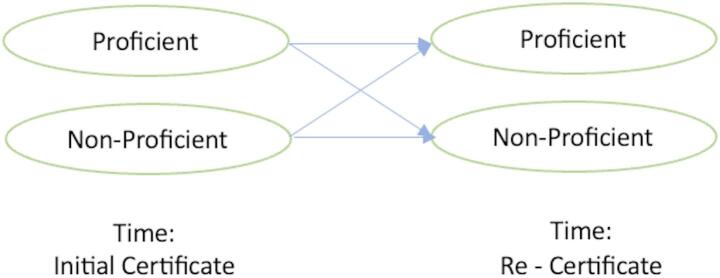
Four possible change patterns

Four possible change patterns are shown in Fig. 1. This is also similar to the mover-stayer model as used in economics or public health context (Albert, 1999).

The latent transition analysis then follows the procedure below:

1. For each time point, a Rasch model is performed to estimate the PAs’ latent proficiency for each system knowledge, and the item parameter is retrieved from the item pool. R package “TAM” is used for the calibration (Robitzsch et al., 2020). For example, if we want the ability of person *p* for neurologic system ($\:{\theta\:}_{p,\:\:neuro,t}$), then all items under this blueprint that this person takes during time point *t* will be used to estimate the latent proficiency of neurologic system.
2. For each time point, respondents (exam takers) are classified into proficient and non-proficient groups for each system knowledge based on their latent proficiency estimated for each respective dimension. For each dimension, the top 60% of the population is deemed proficient, while the rest as non-proficient. The reason we choose 60% is because most medical exam has a relatively high passing rate (e.g., 85%), therefore we deed only the top 60% as the proficient group.
3. Each respondent is then classified into four possible change patterns for each system knowledge.
4. Then, the transition probability is calculated given the occurrence rate of four types of change in the relevant practice profile and system knowledge pairs.

Subsequently, the transition probabilities were compared across different categories to answer the research question.

## Results

Calibration is done, respectively, for each system knowledge for both initial certification and recertification. Since different forms have different numbers of items for each system knowledge, we set the criteria for whether a domain-specific score is reliable: all domain specific scores with a standard error larger than 1 are deemed as not applicable due to insufficient test information for this examinee. As mentioned in previous sections, we calculate the pattern percentage by their respective system knowledge, then we collapse all system knowledge and cluster them by frequency label (1-dominant, 2-relevant, 3-distant). Therefore, we generated the percentage mean estimate by frequency label, as well as the standard error (the variation between different system knowledge divided by the number of effective system knowledge counts). The estimated transition matrices are shown in Table 2. For each frequency label (0- reference, 1-dominant, 2-relevant, 3-distant), the likelihood of all four patterns add to one.

**Table 2:** Estimated transition matrix for system knowledge with different frequency of use

	Number of effective system knowledge count	(0,0)	(0,1)	(1,0)	(1,1)
Reference	Not applicable	0.20	0.25	0.19	0.37
Dominant	12	0.12(0.006)	0.28(0.009)	0.14(0.007)	0.47(0.015)
Relevant	12	0.19(0.008)	0.24(0.008)	0.21(0.007)	0.37(0.018)
Distant	13	0.21(0.004)	0.23(0.003)	0.23(0.002)	0.33(0.003)

Note: Number in parentheses is the standard error of the estimate

According to our hypothesis, the likelihood of maintaining system knowledge (i.e., proficient to proficient, pattern (1,1)) should follow the order: dominant > reference > relevant > distant, since this order reflects the frequency of knowledge being used. Therefore, the likelihood of system knowledge decline (i.e., proficient to non-proficient) should follow the reverse order.

**Fig. 2:**
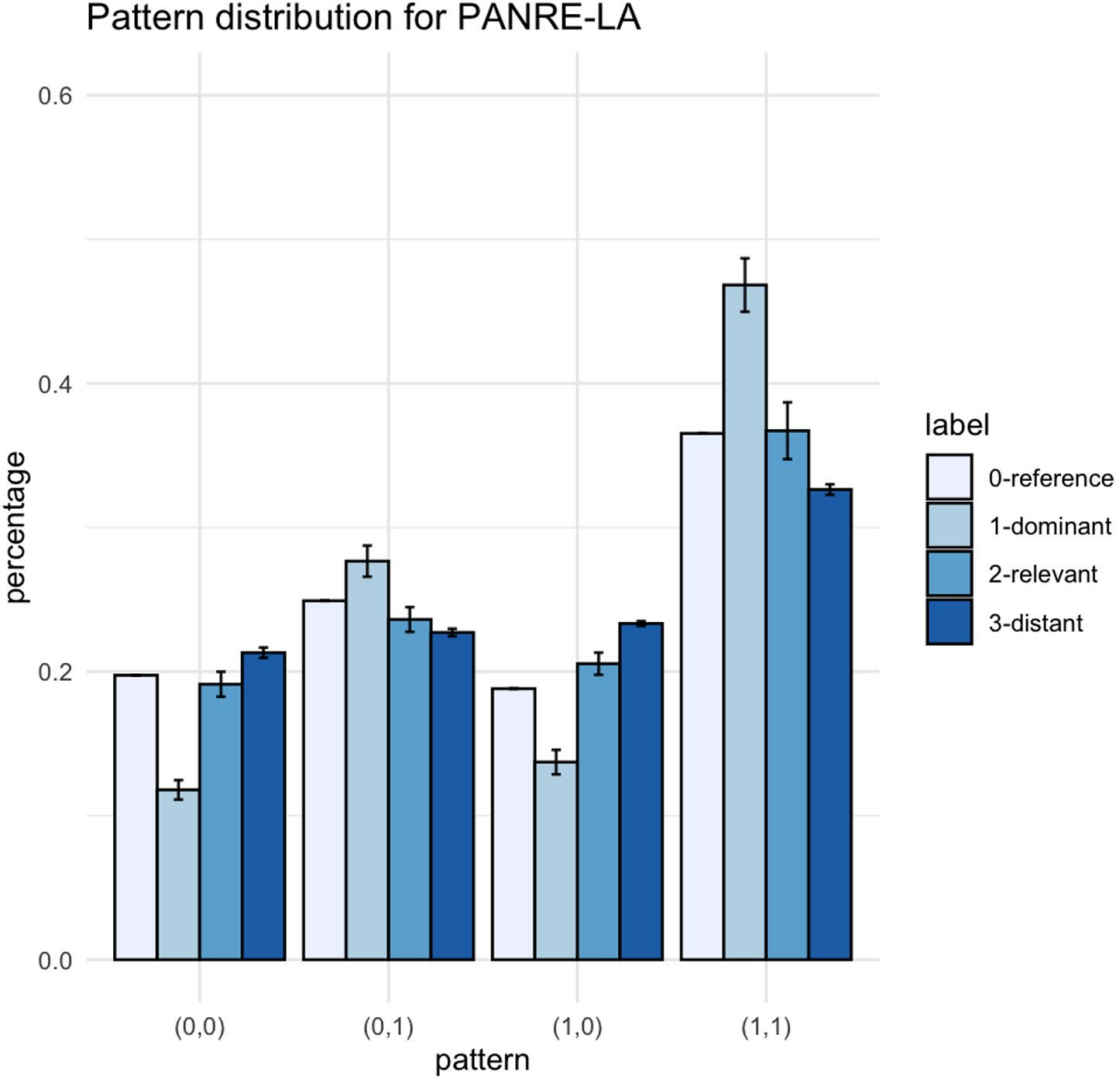
Change pattern distribution between initial certification and recertification

The change pattern is also shown in Fig. 2. First, we investigated the pattern of knowledge decline (i.e., pattern (1,0). Compared to the dominant knowledge, relevant knowledge is more likely to decline (OR = 2.31, CI = [1.82, 2.94], *p* < 0.001), reference knowledge (OR = 1.55, CI = [1.26, 1.91], *p* < 0.001), as well as distant knowledge (OR = 2.26, CI = [1.84, 2.78], *p* < 0.001). Compared to reference knowledge, relevant knowledge and distant knowledge is also more likely to decline (for relevant: OR = 1.49, CI = [1.30, 1.71], *p* < 0.001; for distant: OR = 1.46, CI = [1.36, 1.57], *p* < 0.001). The comparison between relevant and distant is not significant (OR = 0.98, CI = [0.85, 1.12], *p* = 0.73). The order between the first three categories (dominant, reference, relevant) are maintained, therefore, the hypothesis is generally validated by the trend of knowledge decline.

Moreover, we found that the trends of knowledge improvement (i.e., non-proficient to proficient, pattern (0,1)) are also influenced by practice profile, namely, knowledge active in daily use (i.e., dominant knowledge) has higher odds of improving from non-proficient to proficient over time, as compared to relevant (OR *=* 2.19, CI *=* [1.71, 2.81], *p <* 0.001), reference (OR = 1.66, CI = [1.34, 2.05], *p* < 0.001), and distant (OR = 2.12, CI = [1.72, 2.65], *p <* 0.001). Reference knowledge has higher likelihood to improve compared to both relevant and distant (for relevant: OR = 1.32, CI = [1.14,1.53], *p* < 0.001; for distant: OR = 1.29, CI = [1.19,1.39], *p* < 0.001). The comparison between relevant and distant is not significant (OR = 0.97, CI = [0.84, 1.13], *p* = 0.70). In summary, the tendency of knowledge improvement is also impacted by the frequency of knowledge use.

## Discussion

This paper provides the reason why medical knowledge declines after initial certification by comparing the likelihood of different kinds of organ system knowledge decay and draws the conclusion that different kinds of knowledge suffer from various levels of degradation, mitigated by how frequently knowledge was used during PA’s daily practice. Our result shows that the knowledge that is actively being used is less likely to decline, compared to the knowledge that is not being actively used. We found that the rank order of medical knowledge being declined for PAs is: Dominant < Reference < Relevant and Distant. On the other hand, we determined the fact that dominant knowledge is most likely to be retained “proficient” or even “advanced” compared to other type of knowledge (e.g., relevant and distant). Our finding is consistent with Peabody et al. (2014)’s argument, where the authors state that medical practitioners tend to update knowledge in areas that are of greater interest to them. These results inform further testing for recertifying purposes to draw various levels of attention to different kinds of system knowledge. However, our study has certain limitations, one of which is the use of only two time points. This may not adequately capture how the trend evolves over time—for example, whether knowledge continues to decline after re-certification or if the decline eventually reaches a plateau. Further research is needed to explore these patterns, if data permit.

The findings first highlight the importance of mandatory medical knowledge recertification due to observed knowledge decline among practitioners. This decline, which varies by practice profile, raises concerns about maintaining high standards of care. Mandatory recertification helps address this issue, as suggested by Benson (1991). The purpose of recertification is to maintain the high standard of general medical knowledge and ensure the high quality of care the physician provides.

In terms of recertification format, our findings suggest that the areas in which practitioners show weaker performance may vary by specialty, for instance, what constitutes a “dangerous subject” may differ across fields. There is an ongoing debate between maintaining a generalist foundation and tailoring assessments to individual scopes of practice, and given our result, we suggest that adaptive assessment, where the subject is evaluated and tested on those, they had unsatisfactory performance (Weiss & Kingsbury, 1984), may offer a middle ground—ensuring broad competence while also allowing for targeted reinforcement in areas of individual or group weakness. Alternatively, subject test can be supplementary to the general recertification exam.

Lastly, the findings have implications for what should be emphasized during the recertification period (i.e., after initial certification). According to the results, distant knowledge is the area most in need of improvement. Practical settings (e.g., hospitals, clinics, etc.) should devote more attention to this weakness. Moreover, if the assessment system allows for adaptive learning, it could support the targeted reinforcement of domain-specific content. If such efforts lead to consolidation of certain knowledge, they may also help reduce test-taker dissatisfaction—particularly among those who view current assessments as disconnected from overall practice and thus redundant (Lipner et al., 2013).

## Data Availability

The datasets generated and analyzed during the current study are not publicly available due to the confidentiality of individualized data, but de-identified data can be available if requested.

## Footnotes

1
https://www.nccpa.net/become-certified/.

2
https://www.nccpa.net/become-certified/pance-blueprint/.
